# Video-assisted thoracoscopic lobectomy after neoadjuvant chemotherapy for non-small cell lung cancer: a multicenter propensity-matched study

**DOI:** 10.1007/s00464-021-08431-z

**Published:** 2021-03-19

**Authors:** Andrea Dell’Amore, Ivan Lomangino, Nicola Tamburini, Stefano Bongiolatti, Nicola Sergio Forti Parri, William Grossi, Chiara Catelli, Giulia Lorenzoni, Dario Gregori, Samuele Nicotra, Andrea Zuin, Angelo Morelli, Piergiorgio Solli, Luca Voltolini, Giorgio Cavallesco, Federico Rea

**Affiliations:** 1 Thoracic Surgery Unit, Department of Cardiac, Thoracic and Vascular Sciences, University of Padova, Via Giustiniani 2, 35128 Padova, Italy; 2 Thoracic Surgery Unit, Arcispedale Sant’Anna University Hospital, Ferrara, Italy; 3 Thoracic Surgery Unit, Careggi University Hospital, Firenze, Italy; 4 Thoracic Surgery Unit, AUSL Maggiore Teaching Hospital, Bologna, Italy; 5 Thoracic Surgery Unit, Santa Maria della Misericordia University Hospital, Udine, Italy; 6 Unit of Biostatistics, Epidemiology and Public Health, Department of Cardiac, Thoracic and Vascular Sciences, University of Padova, Padova, Italy

**Keywords:** VATS, Non-small-cell lung cancer, Neoadjuvant chemotherapy, Lobectomy, Minimally invasive thoracic surgery

## Abstract

**Background:**

The role of video-assisted thoracoscopic surgery for the treatment of non-small-cell lung cancer after neoadjuvant chemotherapy remains controversial. The aim of this study is to demonstrate the reliability of video-assisted lobectomy compared to the open approach by evaluating perioperative and long-term outcomes.

**Methods:**

In this retrospective, multicentric study from January 2010 to December 2018, we included all patients with non-small-cell lung cancer who underwent lobectomy through the video-assisted or open approach after neoadjuvant chemotherapy. The perioperative outcomes, including data concerning the feasibility of the surgical procedure, the occurrence of any medical and surgical complications and long-term oncological evidence, were collected and compared between the two groups. To minimize selection bias, propensity score matching was performed.

**Results:**

A total of 286 patients were enrolled: 193 underwent thoracotomy lobectomy, and 93 underwent VATS lobectomy. The statistical analysis showed that surgical time (*P* < 0.001), drainage time (*P* < 0.001), days of hospitalization (*P* < 0.001) and VAS at discharge (*P* = 0.042) were lower in the VATS group. The overall survival and disease-free survival were equivalent for the two techniques on long-term follow-up.

**Conclusions:**

VATS lobectomy represents a valid therapeutic option in patients affected by non-small-cell lung cancer after neoadjuvant chemotherapy. The VATS approach in our experience seems to be superior in terms of the perioperative outcomes, while maintaining oncological efficacy.

**Supplementary Information:**

The online version contains supplementary material available at 10.1007/s00464-021-08431-z.

Video-assisted thoracoscopic (VATS) lobectomy is considered the standard treatment for patients with early-stage non-small-cell lung cancer (NSCLC) [1–3]. However, in the case of more advanced NSCLC stages after neoadjuvant multimodality treatment, the feasibility and safety of the VATS technique is still questionable. For VATS pulmonary resection, the presence of adhesions, tissue fragility, delayed healing, fibrosis and tissue edema are the main difficulties encountered after neoadjuvant treatment [4–6]. The aim of this multicentric propensity-score-matched study was to compare the outcomes of patients who received neoadjuvant chemotherapy for NSCLC followed by lobectomy performed though a VATS or open approach.

## Materials and methods

A retrospective multicentric analysis from January 2010 to December 2018 at five Italian thoracic surgery units was performed for patients with NSCLC treated with neoadjuvant chemotherapy who subsequently underwent radical pulmonary resection with lobectomy by thoracotomy or the VATS technique. The Institutional Review Board of each center approved the study, with the requirement for individual patient consent being waived. Patients with multiple primary tumors or tumors other than NSCLC, those who were treated with induction radiotherapy, target therapy or immunotherapy, and those who underwent segmentectomy/wedge resection, pneumonectomy, bilobectomy or sleeve lobectomy were excluded from the study. All patients were staged by means of total body computed tomography (CT) scans, and 18-FDG PET, EBUS-TBNA or mediastinoscopy was used for lymph node evaluation. The neoadjuvant protocols were based on oncologist preference, multidisciplinary discussion and the availability of induction therapy protocols and clinical trials. Only patients with radiological evidence of non-progression of the disease documented by imaging tests performed after the third cycle or at the end of treatment were then eligible for surgery. The surgical technique for VATS lobectomy was previously described, and lateral muscle sparing or posterolateral thoracotomy was used in the open cases [7]. Complete dissection of the hilar and mediastinal lymph node stations was performed in all cases. The conversion from VATS to the open technique was defined by the widening of the anterior access with the use of the rib retractor. Postoperative pain was managed with a peridural catheter or patient-controlled opioid analgesia, in addition to intravenous or oral pain reliever drug therapy. During the hospital stay, pain was measured with the visual analog scale (VAS; from 0 to 10 depending on the patient's subjective perception). The thoracic drainage was removed in the absence of air leaks from the collection system and with liquid leaks of less than 300 ml/24 h. Follow-up data were collected during outpatient visits, and chest CT scans were performed every 6 months for the first two years and then annually. Enhanced recovery after surgery (ERAS) was not in progress in the involved centers during the study period.

### Statistical analysis

Descriptive statistics are reported as quartile I/median/quartile III for continuous variables and percentages (absolute numbers) for categorical variables. A propensity score matching (PSM) approach was employed to account for potential confounding factors related to the non-random allocation of the patients to the two intervention groups. Propensity scores were estimated using the CBPS algorithm. A matching approach was employed using the *k*-nearest neighbor algorithm with a 1:2 ratio and a caliper of 0.25. The postoperative outcome distribution in the intervention groups was evaluated using the Wilcoxon test for continuous variables and the Pearson Chi-squared test for categorical variables. *P*-values were subjected to Benjamini–Hochberg correction to account for the multiplicity of testing. The survival distribution in the intervention groups was evaluated using the Kaplan–Meier approach. The risk of disease relapse and of cancer death were evaluated using cumulative incidence functions (CIFs). The analyses were performed using R-software (version 3.6.2) with the packages Covariate Balancing Propensity Score (CBPS), MatchIt, survey and rms. According to clinical judgment, the baseline variables included in the propensity score estimation were age, sex, BMI (body mass index), diabetes, ischemic heart disease, preoperative FEV1 (forced expiratory volume in the 1st second), DLCO (diffusing capacity for carbon monoxide), smoking history, ASA score, histology, tumor location, TNM (classification of malignant tumors) and N status. The prematching data are reported in the supplemental material section. A Cox proportional hazards model was used to evaluate the effect of “overall-downstaging” on the survival of the matched population. The significance of the predictors was assessed using the likelihood ratio test. The results of the analyses are reported as the hazard-ratio (HR) and 95% confidence interval (95% CI), together with the p-value of the likelihood ratio test. Patients who were converted from VATS to open approach were analyzed in the open group.

## Results

### Patients and treatment characteristics

**Fig.1:**
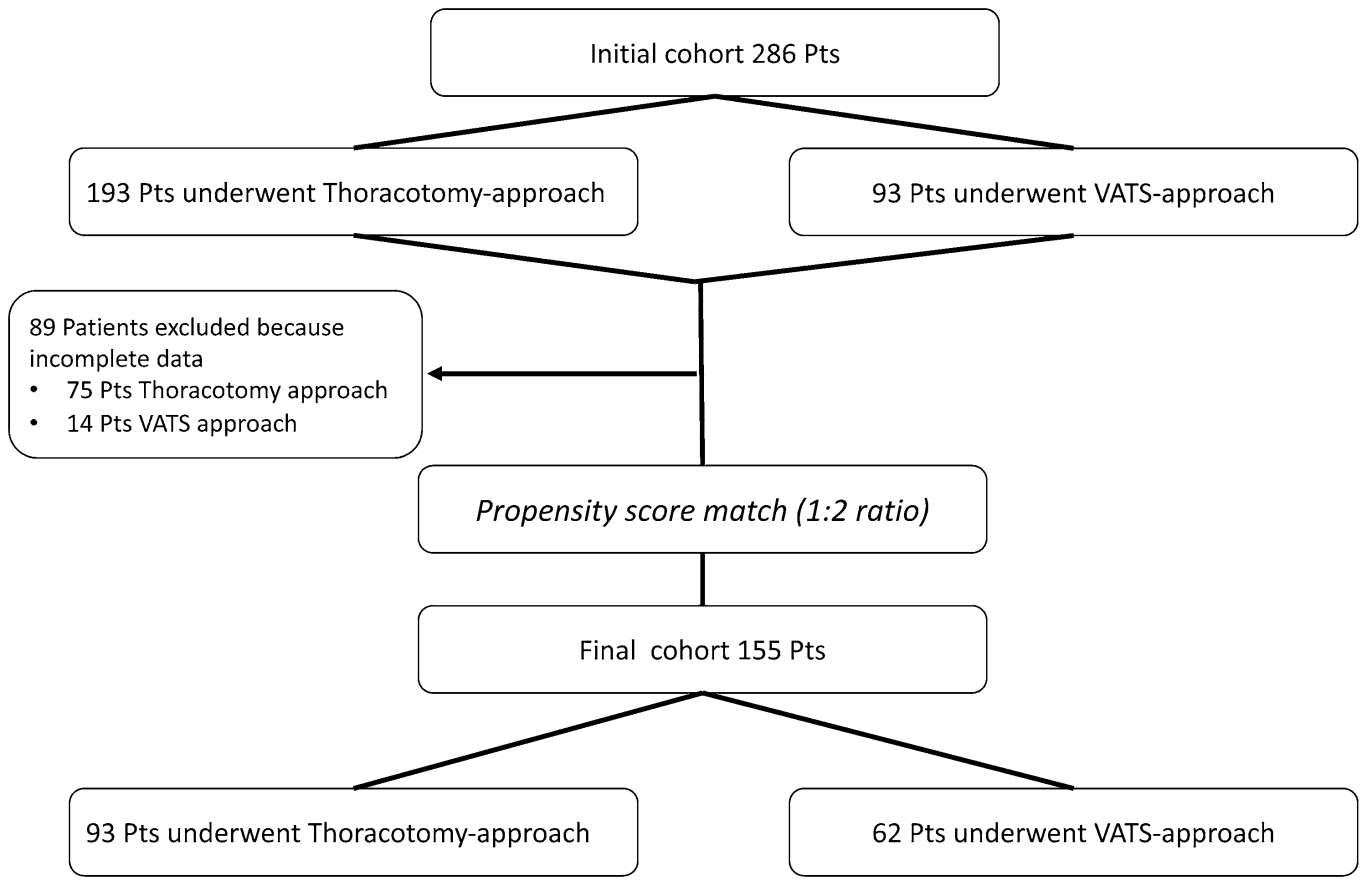
Propensity score matching protocol

The study included a total of 286 patients, of whom 193 underwent thoracotomy lobectomy and 93 underwent VATS lobectomy. After excluding patients with incomplete data, the patients were propensity matched (Fig. 1), achieving a final cohort of 155 patients (93 in the thoracotomy group and 62 in the VATS group). The preoperative population characteristics and operative results before PSM are reported in the supplemental materials. The patient characteristics after the propensity score matching process are shown in Table 1. There were no significant differences in the baseline data between the two groups. The neoadjuvant regimens with the number of administered cycles are summarized in Table 2. Table 1 shows the oncological clinical staging of both groups. The majority of patients were in stage IIIa (76%), and cN2 disease was reported in 65% of the patients. The patients with clinical stage IIa (three patients) and several patients in IIb (five patients) received preoperative chemotherapy because they initially refused surgery or because they were enrolled in a clinical trial. There was no difference between the groups in terms of neoadjuvant treatment, histology or staging after PSM. The conversion rate from VATS to thoracotomy was 8.6% (eight patients). These patients were analyzed among the thoracotomy group patients. The reasons for conversion were bleeding (three patients), metastatic lymph nodes (four patients) and severe adhesions (one patient). All the matched patients received a complete R0 resection.

**Table 1:** Neoadjuvant chemotherapy regimens

Therapy regimens	%	VATS	%	Open
		*n* cycles		*n* cycles
Carboplatin + Gemcitabine	20	4	16	4
Carboplatin + Paclitaxel	6	8	1	8
Carboplatin + Pemetrexed	17	4	9	4
Carboplatin + Docetaxel	3	3	0	0
Carboplatin + Taxol	3	5	1	4
Cisplatin	0	0	1	6
Cisplatin + Gemcitabine	20	3	24	4
Carbo/Cisplatin + Gemcitabine + Taxol	3	8	13	3
Cisplat. + Gemcitab.; Pemetrexed; Carboplatin + Pemetrexed	0	0	1	15
Cisplatin + Pemetrexed	17	4	19	4
Cisplatin + Taxol	0	0	1	3
Cisplatin + Vinorelbine	0	0	3	3
Vinorelbine + Docetaxel	0	0	1	3
Cisplatin + Etoposide	3	3	0	0
Cisplatin + Docetaxel	3	3	0	0
Unknown	6		9

**Table 2:** Preoperative patient’s characteristics after PSM

Variables	Open (*N* = 93)	VATS (*N* = 62)	Combined (*N* = 155)	*P*-value
Age	62/68/72	58/69/74	60/68/72	0.662
Gender				0.786
Male	62% (58)	65% (40)	63% (98)	
Female	38% (35)	35% (22)	37% (57)	
BMI(Kg/m2)	26 (IQR 23–29)	25 (IQR 23–28)	25 (IQR 23–28)	0.605
Diabetes	15% (14)	16% (10)	15% (24)	0.856
Chronic renal failure	4% (4)	6% (4)	5% (8)	0.3
Polivasculopathy	41% (38)	53% (33)	46% (71)	0.4
Hypertension	59% (55)	47% (29)	54% (84)	0.1
History of malignancies	18% (17)	18% (11)	18% (28)	0.6
COPD	15% (14)	16% (10)	15% (24)	0.609
Ischemic heart disease	19% (18)	16% (10)	18% (28)	0.902
FEV1(%)	90 (IQR 77–101)	95 (IQR 82–103)	91 (IQR 80–102)	0.238
DLCO(%)	71 (IQR 60–84)	76 (IQR 59–81)	73 (IQR 60–83)	0.763
Smoking history				0.698
Never	17% (16)	19% (12)	18% (28)	
Previous	27% (25)	21% (13)	25% (38)	
Active	56% (52)	60% (37)	57% (89)	
Pack year	40 (IQR 21–52)	35 (IQR 20–43)	40 (IQR 20–50)	0.2
ASA > 3	35% (33)	35% (22)	35% ( 55)	1
Central tumor	34% (32)	34% (21)	34% (53)	0.945
Peripheral tumor	66% (61)	66% (41)	66% (102)	
cTNM 8th				0.643
Stage IIa + b	11% (10)	6% (4)	9% (14)	
Stage IIIa	74% (69)	79% (49)	76% (118)	
Stage IIIb	15% (14)	15% (9)	15% (23)	
cN disease				0.586
N0	8% (7)	8% (5)	8% (12)	
N1	30% (28)	23% (14)	27% (42)	
N2	62% (58)	69% (43)	65% (101)	

*IQR* interquartile range, *BMI* body mass index, *COPD* chronic obstructive pulmonary disease, *FEV1* forced expiratory volume in 1 s, *DLCO* diffusion lung carbon monoxide

### Perioperative outcomes

All the variables considered for the perioperative outcomes are shown in Table 3. The overall in-hospital mortality was 1.3% (2/155 patients), without a difference between the two groups. The analysis revealed statistically significant values for the VATS approach with respect to the open approach in terms of surgical time (*P* < 0.001), drainage days (*P* < 0.001) and the VAS at discharge (*P* = 0.042). The total number of lymph nodes removed was higher in the open group (*P* = 0.02), but there was no difference in terms of station numbers (*p* = 0.6). The ICU (intensive care unit) time (*P* = 0.615) was not significant. A total of 23 adverse events occurred in 17 patients in the open group, and 14 adverse events occurred in eight patients in the VATS group. The postoperative surgical complications (bleeding, prolonged air leaks, pneumothorax, pleural effusion and chylothorax), which were the lowest in the VATS group, were not statistically significant (*P* = 0.2). In detail, prolonged air leaks were reported in 19 patients after open surgery (21%) and in 11 patients after VATS surgery (17%) (*p* = 0.064). Medical complications (atrial fibrillation, atelectasis, pneumonia, myocardial infarction, renal failure and pulmonary embolism) were significantly lower in the VATS group (*p* = 0.05). VATS lobectomy was associated with a shorter length of hospital stay (*p* < 0.01). Because of the advanced tumor stages in most of the patients, adjuvant chemotherapy was recommended in 40 patients (19 in the open group and 21 in the VATS group). Adjuvant chemotherapy protocols were similar and not influenced by either the VATS or open approach. In analyzing the time between surgery and the commencement of chemotherapy, an earlier start of therapy administration was found for the VATS group (48 days for the open group vs 32 days for the VATS group, *p* = 0.09). The open group received more adjuvant radiotherapy (*p* = 0.002). The oncologic results and treatments are summarized in Table 4.

**Table 3:** Intraoperative and perioperative characteristics

Variables	Open (*N* = 93)	VATS (*N* = 62)	Combined (*N* = 155)	*P*-value
Surgical time (min)	185 (IQR 160–210)	158 (IQR 125–200)	180 (IQR 140–210)	< 0.001
Resected lymph nodes (*n*)	26 (IQR 16–36)	20 (IQR 14–27)	24 (IQR 15–30)	0.022
Nodes station (*n*)				0.6
N1 station	3.0 (IQR 2–4)	3.2 (IQR 2–4)		
N2 station	4.4 (IQR 3–5)	4.7 (IQR 3–5)		
Postoperative-ICU				0.546
No	71% (66)	65% (40)	68% (106)	
Yes	29% (27)	35% (22)	32% (49)	
ICU-stay (h)	25.3 (IQR 24–55)	24.8 (IQR 24–24)	24 (IQR 24–42)	0.615
Chest tube duration	4 (IQR 4–5)	2 (IQR 2–4)	4 (IQR 3–5)	0.018
In-hospital stay (days)	8 (IQR 7–12)	6 (IQR 5–8)	8 (IQR 6–10)	0.01
Postoperative surgical complications				0.219
No	80% (74)	88% (55)	83% (129)	
Yes	20% (19)	12% (7)	17% (26)	
Postoperative medical complications				0.054
No	83% (77)	94% (58)	86% (133)	
Yes	17% (16)	6% (4)	14% (22)	
VAS (discharge)	3 (IQR 2–5)	2 (IQR 0–3)	2 (IQR 0–4)	0.042

*IQR* interquartile range, *ICU* intensive care unit, *VAS* analogue visual pain scale

**Table 4:** Oncological results and adjuvant treatments

Variables	Open (*N* = 93/91)*	VATS (*N* = 62)	Combined (*N* = 155/153)*	*p*-value
Histology				0.6
Adenocarcinoma	69% (64)	72% (45)	71% (109)	
Squamous cell carcinoma	23% (21)	20% (12)	21% (33)	
Other	9% (8)	8% (5)	8% (13)	
pTNM-stage				0.3
CR(T0N0)	1% (1)	1.6% (1)	1.3% (2)	
Ia	11% (10)	10% (6)	9% (14)	
Ib	11% (10)	18% (11)	8% (12)	
IIa	11% (10)	16% (10)	13% (20)	
IIb	15% (14)	18% (11)	17% (26)	
IIIa	32% (30)	31% (19)	30% (46)	
IIIb	15% (14)	23% (14)	19% (30)	
IIIc	2% (2)	–		
IV	1% (1)	3% (2)	2% (3)	
pN-disease				0.6
pN0	45% (42)	44% (27)	45% (69)	
pN1	16% (15)	18% (11)	17% (26)	
pN2	37% (34)	39% (24)	37% (58)	
pN3	2% (2)	–	1% (2)	
Overall downstaging	40% (37)	45% (28)	42% (65)	0.6
Adjuvant chemotherapy	21% (19)	34% (21)	26% (40)	0.07
Time from surgery to adjuvant therapy(days)	48 (IQR 32–61)	32 (IQR 30–40)	39 (IQR 30–44)	0.09
Cycles numbers	3 (IQR 2–4)	4 (IQR 3–5)	4 (IQR 2–4)	0.08
Suspension for toxicity	26% (5)	19% (4)	22% (9)	0.059
Adjuvant radiotherapy	56% (51)	31% (19)	46% (70)	0.002
Immunotherapy	1% (1)	1.6% (1)	1% (2)	0.08
Target therapy	9% (8)	8% (5)	9% (13)	0.5
Recurrence				0.1
Loco-regional	11% (10)	13% (8)	4% (26)	
Systemic	49% (45)	42% (26)	46% (71)	
Median recurrence time (months)	16.8 (IQR 8.4–40)	18 (IQR 12–28)	18(IQR 8–34)	0.9

*IQR* interquartile range, *CR* complete response

### Survival analysis

**Fig. 2:**
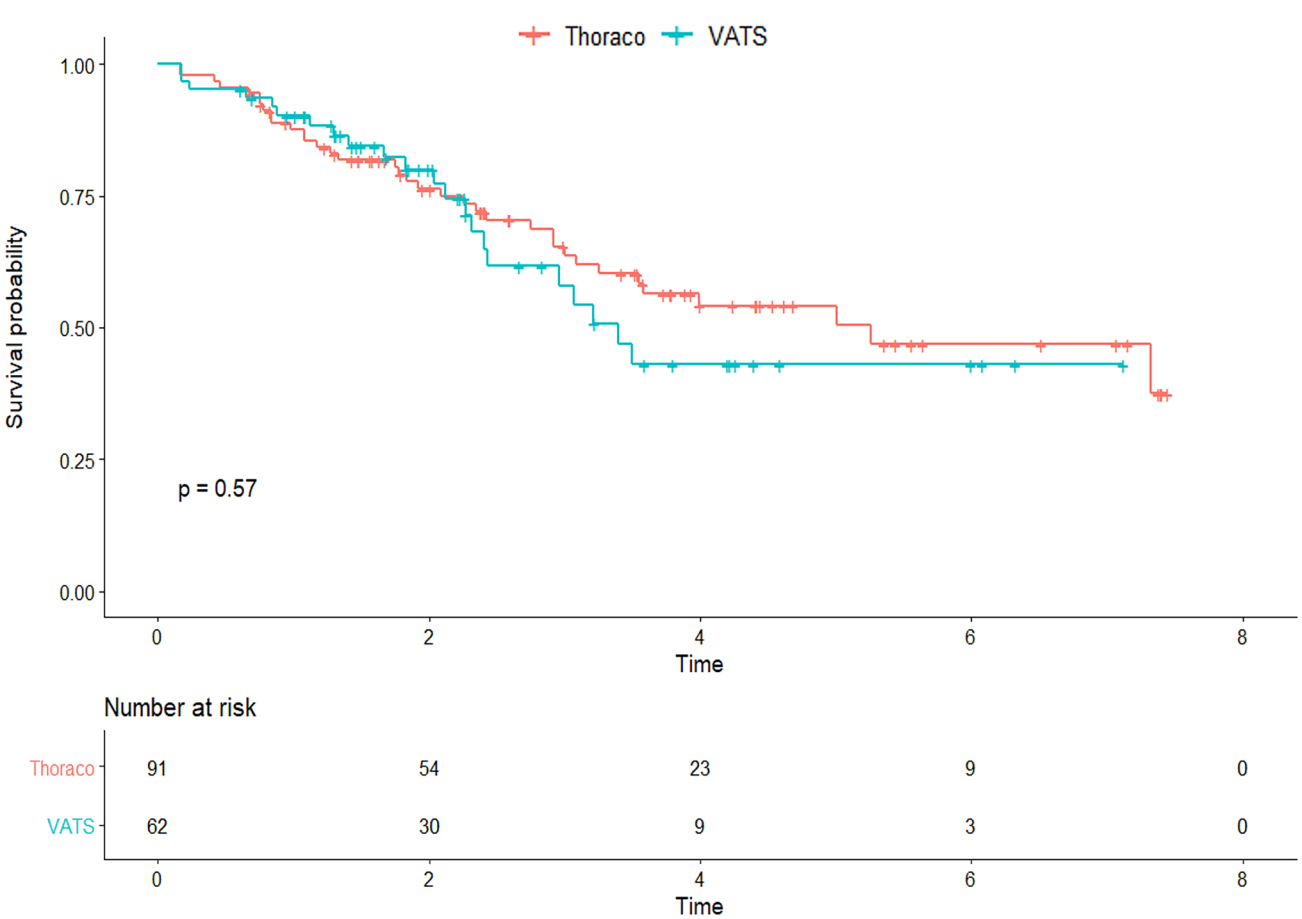
Overall survival stratified for the two surgical approaches

**Fig. 3:**
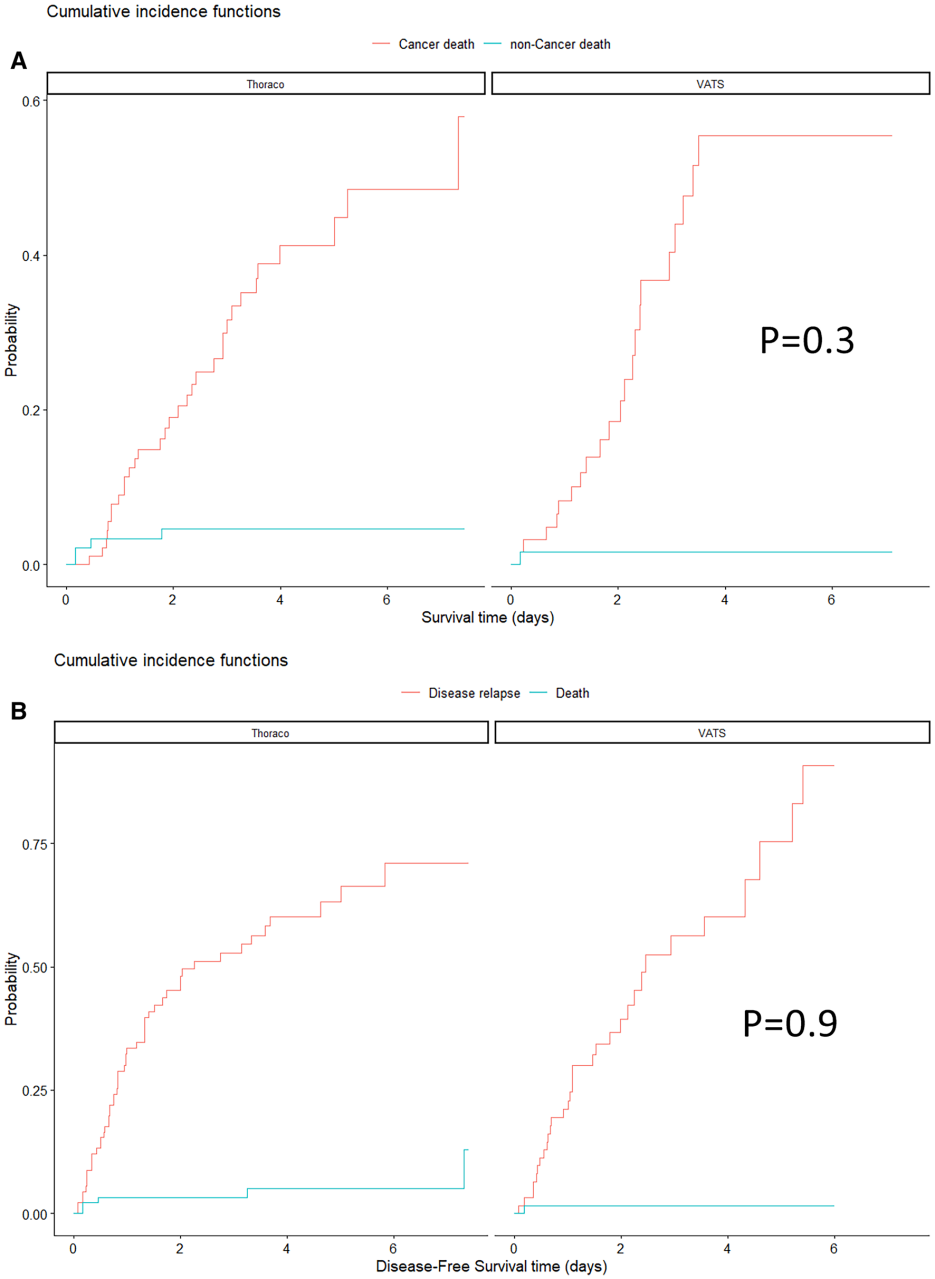
**A** Comparison between the two techniques of stratified cumulative incidence analysis for cancer-related and non-cancer-related death. **B** Cumulative incidence of disease-free survival

The median follow-up time was 68 months (IQR 40–111). Overall survival at 1, 3 and 5 years was not significantly different between the open and VATS groups: 1-year survival 88% and 90%, 3-year survival 64% and 58% and 5-year survival 54% and 43%, respectively (*p* = 0.6) (Fig. 2). The analysis of the cumulative incidence, which compared the cancer-related deaths and the non-cancer-related deaths (Fig. 3A) of the two surgical approaches, confirmed the absence of a difference between the groups. Similarly, disease-free survival (DFS) was not significantly different between the open and VATS groups: 1-year DFS 67% and 77%, 3-year DFS 47% and 44% and 5-year DFS 37% and 25%, respectively (Fig. 3B). Tumor downstaging was associated with better survival in the regression analysis (*p* = 0.001), independent of the surgical approach.

## Discussion

In the early nineties, the introduction of VATS lobectomy was debated and opposed by many for a supposed lack of safety, feasibility and poor oncologic efficacy. This philosophy was disproved over the following years. Currently, the effectiveness of VATS in terms of a lower morbidity rate, less postoperative pain, shorter hospitalization and faster recovery with optimal oncologic results in early-stage NSCLC has been clearly demonstrated [1–3, 8, 9]. The use of VATS has been introduced in recent years for the surgical treatment of locally advanced NSCLC [10, 11]. However, several concerns regarding oncologic results, technical challenges and postoperative morbidity and mortality have limited the use of VATS when dealing with more advanced NSCLC stages, particularly after neoadjuvant treatment. The main limitations of these studies are the limited number of enrolled patients, the different multimodal protocols applied over the years and selection biases due to the surgeon’s preference, skills and experience as well as the patient’s preoperative selection of the VATS or open approach. We performed a multicentric study of different thoracic surgery departments that shared the same surgical approaches together with VATS experience and skills. Moreover, we considered only patients treated in recent decades with the aims of limiting the effects of induction therapy, anesthesiology management, surgical techniques and instrumentation evolution during the “eras”. At the same time, we omitted the first part of the VATS surgery learning curves during the nineties, when VATS was more of a pioneering technique than a gold standard compared to open surgery. Afterwards, to flatten the patient’s selection bias, we performed propensity score matching, achieving a well-balanced cohort of patients in all the analyzed categories between VATS and open lobectomy. We included in the study only lobectomies performed after chemotherapy to avoid further confounding factors.

Regarding the surgical feasibility of VATS lobectomy after neoadjuvant therapy, an indicator to take into account is the length of the operating time, which expresses the difficulty and feasibility of the surgical steps of adherence lysis, isolation of vascular elements and lymph node dissection. Our study showed a lower operative time in the VATS group (*p* < 0.001). The conversion from VATS to open surgery, an indicator also useful for assessing the safety of the procedure, was carried out in eight patients (8.6%). The reasons for this conversion were the difficulty of performing a complete lymphadenectomy of metastatic nodes, with the risk of bleeding complications. In the literature, the conversion rate for VATS lobectomy for early-stage NSCLC is between 7 and 23% [1–3, 8, 9]. In a meta-analysis of VATS lobectomy in early-stage NSCLC, Yan et al. [3] reported a median conversion rate of 8.1%. Thus, our study confirmed that the risk of conversion during VATS lobectomy in patients after induction chemotherapy is not higher than that in non-chemotherapy-treated patients.

Regarding the hospital stay, our results confirmed the superiority of the VATS technique compared to thoracotomy in postoperative outcomes. In fact, postoperative pain, expressed by the VAS at the time of discharge, and even the days of permanence of the pleural drainage were significantly lower in the VATS group, thus leading to a significant reduction in the hospital stay, which was already well established by countless similar studies [1–3, 9]. The incidence of postoperative surgical complications between the two groups was not significantly different. In particular, prolonged air leakage (> 5 days) occurred with a slightly greater frequency in the open lobectomy group (20% in the open group versus 17% in the VATS group) but without reaching statistical significance (*p* = 0.06). On the other hand, postoperative medical complications, mainly due to the appearance of supraventricular arrhythmias and atelectasis, presented a lower frequency in the VATS group. The reported overall complication rate after VATS lobectomy for early-stage NSCLC ranged between 10 and 21%. We reported a 16% overall complication rate in the VATS group, confirming that it does not differ from the post-chemotherapy VATS lobectomy morbidity rate [1–3, 8–10]. It has been postulated in the literature that after VATS surgery, decreased morbidity, shorter hospital stays and faster patient recovery could result in a shorter interval between surgery and adjuvant therapy and that the patients endure chemotherapy toxicity better. Petersen et al. found a higher compliance to start adjuvant chemotherapy with less delay after VATS surgery [11]. Similar results were also reported by Teh et al. [12], with a shorter interval between surgery and chemotherapy in the VATS group (55 vs 67 days, *p* = 0.046). In the propensity-score-matched study published by Lee et al. [13], a higher proportion of patients were able to tolerate full cycles of chemotherapy after VATS. We investigated these aspects and found that the time between surgery and chemotherapy was lower in the VATS group, but without reaching statistical significance (*p* = 0.09). These data are difficult to interpret, as they can be influenced by organizational aspects and the interregional mobility of patients in Italy, in addition to the oncologists and patient preference; thus, they should be interpreted with caution. None of our patients had chemotherapy precluded because of postoperative conditions, regardless of the group. Moreover, the VATS group patients seemed to tolerate the full planned cycles and dose better than the open group patients, but this trend was not statistically significant (*p* = 0.08 and *p* = 0.06, respectively). Teh et al. [12] reported a trend of lower toxicity in the VATS group than in the open group without statistical significance, as in our study. All the patients underwent systematic lymphadenectomy, and the median number of removed lymph nodes per patient was in favor of the open group when compared to the VATS group (*p* < 0.002). Despite these differences, our long-term results showed the same oncologic efficacy between VATS and thoracotomy. Overall survival was unchanged by comparison, also in stratifying survival by cancer-related death and non-cancer-related death. Regarding disease-free survival, no statistically significant differences between the two groups were evident. In the literature, several studies reported comparable long-term oncological results between VATS and open surgery after chemotherapy for early-stage NSCLC as well [14–18]. Yang et al. [19] reported a trend toward better 3-year survival in the VATS group, which was completely balanced after propensity score matching. Naturally, patients who had an overall downstaging after neoadjuvant chemotherapy showed better long-term survival and lower recurrence rates. Voltolini et al. reported a 3-year survival of 59% compared to 0% in NSCLC stage IIIa (N2) patients after neoadjuvant treatment with demonstrated downstaging of the disease [20]. This probably reflects the section of “winner patients” and is clearly not influenced by the surgical approach. Nevertheless, in our experience, patients who had a very evident response to chemotherapy with important reduction of the T and N factors sometimes develop very dense and fibrotic/inflammatory tissue around the regressed tumor/nodes, making the VATS approach more challenging.

In the future, the use of targeted therapy and immunotherapy could become useful strategies for neoadjuvant treatment. These new therapies could influence the surgical results and, as already reported from small case series, particularly after immunotherapy, tissue quality, adhesions, inflammation and post-therapy fibrosis seem to be much worse than those in the case of standard platinum-based treatment [21]. The role of VATS surgery in these cases needs to be evaluated by further studies. In our study, none of the patients had targeted or neoadjuvant immunotherapy, which could be considered a further limitation of this study from a future perspective for VATS surgery.

## Conclusions

Compared to the open technique, the minimally invasive technique for patients with NSCLC after neoadjuvant chemotherapy seems to be superior in terms of the perioperative outcomes, allowing patients to achieve a faster physical recovery. The oncological efficacy in terms of global survival and disease-free survival was not inferior to that of open surgery. Therefore, VATS lobectomy represents a valid therapeutic option in patients with locally advanced neoplastic disease after neoadjuvant chemotherapy.

## Supplementary Information

Below is the link to the electronic supplementary material.

Supplementary file 1 (DOCX 23 kb)
