# Supplementary material for: Video-assisted thoracoscopic lobectomy after neoadjuvant chemotherapy for non-small cell lung cancer: a multicenter propensity-matched study
Source: Surg Endosc. 2021 Mar 19;36(2):1466–75. doi: 10.1007/s00464-021-08431-z (PMC8758629; doi:10.1007/s00464-021-08431-z)
Supplement: Supplementary file 1 — (DOCX 23 kb) [file 464_2021_8431_MOESM1_ESM.docx]

| **Characteristics** | **OPEN (N=193)** | **VATS (N=93)** | **Combined (N=286)** | **P-value** |
| --- | --- | --- | --- | --- |
| Age(years) | 67(IQR60-71) | 68(IQR59-74) | 68(IQR60-72) | 0.413 |
| Gender  Male  Female | 64%(124)  36%(69) | 59% (55)  41% (38) | 63% (179)  37% (107) | 0.403 |
| BMI | 25(IQR 23-29) | 25(IQR23-28) | 25(IQR23-29) | 0.528 |
| Diabetes | 89% (171) | 82% (76) | 86% (247) | 0.112 |
| COPD | 22% (43) | 17% (16) | 21% (59) | 0.31 |
| Chronic Renal Dysfunction | 2% (3) | 6% (6) | 3% (9) | 0.026 |
| Hypertension | 38% (72) | 48% (45) | 41% (117) | 0.08 |
| Peripheral Vascular Disease | 8% (16) | 12% (11) | 9% (27) | 0.338 |
| Ischemic heart disease | 5% (9) | 10% (9) | 6% (18) | 0.102 |
| FEV1 (%) | 88(IQR75-100) | 94(IQR83-104) | 90(IQR77-102) | 0.017 |
| DLCO (%) | 68(IQR55-83) | 74(IQR57-81) | 70(IQR56-82) | 0.237 |
| Smoking History  Never  Previous  Active | 28% (54)  42% (81)  30% (58) | 22% (20)  53% (49)  26% (24) | 26% (74)  45% (130)  29% (82) | 0.223 |
| Pack year | 40 (IQR20-51) | 35(IQR20-43) | 40(IQR20-50) | 0.179 |
| ASA>3 | 31% (59) | 30% (28) | 30% (87) | 0.937 |
| History of Malignancies | 13% (25) | 15% (14) | 14% (39) | 0.628 |
| Histology  Adenocarcinoma  Squamous cell carcinoma  Others | 68% (131)  23% (45)  9% (17) | 85% (79)  10% (9)  5% (5) | 73% (210)  19% (54)  8% (22) | 0.008 |
| Central tumor | 36% (69) | 31% (29) | 35% (98) | 0.411 |
| Peripheral tumor | 64% (122) | 69% (64) | 65% (186) |  |
| cTNM 8th  Stage IIa+b  Stage IIIa  Stage IIIb | 12% (24)  12% (23)  76% (146) | 5% (5)  14% (13)  81% (75) | 10% (29)  13% (36)  77% (221) | 0.174 |
| cN disease  N0  N1  N2 | 7% (13)  31% (59)  63% (121) | 5% (5)  27% (25)  68% (63) | 6% (18)  29% (84)  64% (184) | 0.696 |
| IQR: interquartile range, BMI: body-max-index, COPD: chronic obstructive pulmonary disease, FEV1: forced expiratory volume in 1-second, DLCO: Diffusion lung carbon monoxide, | | | | |

**Table.1 supplemental:** preoperative patient’s characteristics before propensity score matching

**Table.2 supplemental:** intraoperative and perioperative characteristics before propensity score matching

| **Characteristics** | **OPEN (N=193)** | **VATS (N=93)** | **Combined (N=286)** | **P-value** |
| --- | --- | --- | --- | --- |
| Tumor Size (cm) | 4.7(IQR3-6.7) | 3.6(IQR2.9-5) | 4.1(IQR3-6) | 0.002 |
| Post-chemotherapy tumor size (cm) | 4(IQR42.5-5) | 3(IQR2-4) | 3.6(IQR2.2-4.5) | 0.003 |
| pTNM 8th  CR  Ia  Ib  IIa  IIb  IIIa  IIIb  IIIc  IV | 1% (2)  8% (16)  10% (19)  11% (21)  24% (46)  27% (52)  16% (30)  1% (2)  3% (5) | 2% (2)  6% (6)  4% (4)  12% (11)  16% (15)  38% (35)  14% (13)  1% (1)  6% (6) | 2% (5)  8% (22)  8% (23)  11% (31)  21% (61)  30% (87)  15% (43)  1% (3)  4% (11) | 0.085 |
| pN disease  N0  N1  N2  N3 | 47% (90)  23% (44)  30% (57)  1% (2) | 38% (35)  22% (20)  40% (37)  1% (1) | 44% (125)  22% (64)  33% (94)  1% (3) | 0.359 |
| Completeness of the resection  R0  R1 | 179(93%)  14(7%) | 89(96%)  4(4%) | 268(94%)  18(6%) | 0.083 |
| Overall Down Staging | 49% (95) | 42% (39) | 47% (134) | 0.247 |
| Lymph Nodes (N) Down Staging | 53% (103) | 46% (43) | 51% (146) | 0.258 |
| Postoperative Surgical complications | 24%(46) | 16%(15) | 21%(61) | 0.15 |
| Postoperative medical complications | 23%(45) | 8%(7) | 18%(52) | 0.003 |
| VAS (discharge) | 3(IQR2-5) | 1(IQR0-3) | 3(IQR1-4) | 0.003 |
| Reintubation | 4% (7) | 1% (1) | 3% (8) | 0.22 |
| Tracheotomy | 1% (2) | 0% (0) | 1% (2) | 0.325 |
| Surgical Hemothorax | 4% (7) | 1% (1) | 3% (8) | 0.22 |
| Postoperative Transfusion | 11% (21) | 1% (1) | 8% (22) | 0.004 |
| Discharge with Heimlich Valve | 4% (7) | 2% (2) | 3% (9) | 0.503 |
| In Hospital Mortality | 2% (4) | 0% (0) | 1% (4) | 0.162 |
| Time from surgery to adjuvant therapy(days) | 46(IQR35-57) | 32(IQR30-39) | 39(IQR30-49) | 0.004 |
| Adjuvant Chemotherapy | 22% (41) | 38% (35) | 27% (76) | 0.005 |
| Adjuvant Target Therapy | 69% (11) | 29% (10) | 42% (21) | 0.009 |
| Adjuvant Immunotherapy | 25% (3) | 3% (1) | 9% (4) | 0.022 |
| Adjuvant Radiotherapy | 39% (74) | 34% (31) | 37% (105) | 0.429 |
| Follow-up Time (days) | 880(IQR54-1433) | 737(IQR434-1084) | 827(IQR489-1332) | 0.008 |
| Recurrence Time (days) | 597(IQR279-1218) | 536(IQR252- 870) | 561(IQR273-1098) | 0.265 |
| IQR: interquartile range, ICU: intensive care unit, VAS: analogue visual pain scale. CR: complete response | | | | |
